# A multi‐centered prospective birth cohort study in Western China

**DOI:** 10.1002/imt2.70049

**Published:** 2025-05-26

**Authors:** Xiangyu Li, Ying Wu, Bin Yi, Mengjie Chen, Gang Zhang, Xiaoshan Shao, Xiulian Jiang, Yuxia Cui, Li Chen, Xiaojing Dong, Shu Zhang, Yao Zhao, Yuebi Deng, Xueqi Li, Yang Wang, Lei Wu, Yu Fu, Dan Ran, Chen Peng, Xiao Yang, Lan Zhang, Yanxia Wang, Yi Zhu, Dina Sun, Yuchen Ran, Dan Zheng, Xuan Yin, Yufen Chen, Yu Long, Wenjing Wang, Xiaodong Zhao, Enmei Liu, Tao Xu, Qiu Li, Wen Zhong

**Affiliations:** ^1^ Department of Early Life Development and Cohort Research Laboratory Children's Hospital of Chongqing Medical University, National Clinical Research Center for Child Health and Disorders, Ministry of Education Key Laboratory of Child Development and Disorders, Chongqing Municipal Health Commission Key Laboratory of Children's Vital Organ Development and Diseases Chongqing China; ^2^ Guangzhou National Laboratory Guangzhou China; ^3^ Chengdu Women's and Children's Central Hospital, School of Medicine University of Electronic Science and Technology of China Chengdu China; ^4^ Gansu Provincial Maternity and Child‐Care Hospital (Gansu Provincial Central Hospital), Gansu Provincial Pediatric Clinical Research Center Lanzhou China; ^5^ The Second Affiliated Hospital of Chongqing Medical University Chongqing China; ^6^ Department of Obstetrics Sichuan Provincial Women's and Children's Hospital, The Affiliated Women's and Children's Hospital of Chengdu Medical College Chengdu China; ^7^ Department of Hepatobiliary and Vascular Surgery The First Affiliated Hospital of Chengdu Medical College Chengdu China; ^8^ Department of Renal Rheumatology and Immunology Guiyang Maternal and Child Health Care Hospital Guiyang China; ^9^ Qinghai Provincial Women and Children's Hospital Xining China; ^10^ Shanghai Children's Medical Center Guizhou Hospital Shanghai Jiao Tong University School of Medicine Guiyang China; ^11^ Growth, Development and Mental Health Center of Children and Adolescents, Chongqing Key Laboratory of Child Neurodevelopment and Cognitive Disorders, National Clinical Research Center for Child Health and Disorders, Ministry of Education Key Laboratory of Child Development and Disorders Children's Hospital of Chongqing Medical University Chongqing China; ^12^ Department of Child Healthcare Sichuan Provincial Women's and Children's Hospital, The Affiliated Women's and Children's Hospital of Chengdu Medical College Chengdu China; ^13^ Department of Maternity Health Guiyang Maternal and Child Health Care Hospital Guiyang China; ^14^ Department of Rheumatism and Immunology Children's Hospital of Chongqing Medical University, National Clinical Research Center for Child Health and Disorders, Ministry of Education Key Laboratory of Child Development and Disorders, Chongqing Key Laboratory of Child Rare Diseases in Infection and Immunity Chongqing China; ^15^ Department of Respiratory Medicine Children's Hospital of Chongqing Medical University, National Clinical Research Center for Child Health and Disorders, Ministry of Education Key Laboratory of Child Development and Disorders, Chongqing Key Laboratory of Child Rare Diseases in Infection and Immunity Chongqing China; ^16^ Department of Nephrology Children's Hospital of Chongqing Medical University, National Clinical Research Center for Child Health and Disorders, Ministry of Education Key Laboratory of Child Development and Disorders, Chongqing Key Laboratory of Pediatric Metabolism and Inflammatory Diseases Chongqing China

## Abstract

The Western China Birth Cohort (WCBC) is a large‐scale, multi‐centered, prospective birth cohort study designed to address critical gaps in maternal and child health research in Western China, a region with diverse altitudes, ethnic groups, and unique environmental exposures. WCBC had enrolled 15,093 pregnant women across eight clinical centers in five provinces (Qinghai, Gansu, Guizhou, Chongqing, and Sichuan), spanning from the high‐altitude Qinghai‐Tibet Plateau to lowland regions. WCBC has collected over 220,000 medical records, 80,000 questionnaires, and 12 different types of biological samples, including peripheral blood, cord blood, dried blood spots, placenta, umbilical cord, decidua, saliva, feces, throat and nasal swabs, vaginal swabs, and breast milk. By integrating advanced multi‐omics measurement, including genomics, proteomics, exosome profiling, metabolomics, spatial transcriptomics, single‐cell RNA sequencing, culturome, metagenomics, and virosome analysis, WCBC provides a valuable platform to explore gene‐environment interplay, early‐life determinants of health, and long‐term disease risks in diverse populations in Western China.

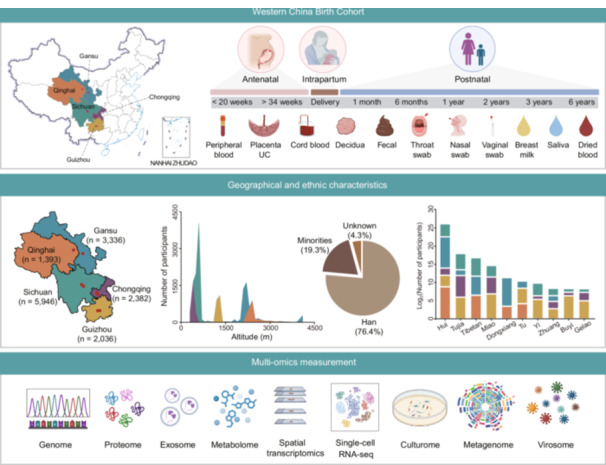

## ETHICS STATEMENT

Ethics approval (No. 4‐5/2020 and 260‐1/2023) was obtained from the ethics committee of Children's Hospital of Chongqing Medical University and other participating hospitals. Written informed consent was obtained from all participants before enrollment. All procedures involving human participants were conducted in accordance with the principles outlined in the Declaration of Helsinki.

To the Editor,

The perinatal and early childhood development periods are critical for human lifelong health, where a wide range of biological, environmental, and social determinants can impact fetal growth, birth outcomes, and future disease risks [1]. Birth cohorts, by longitudinally tracking individuals from the earliest stages of life, provide comprehensive insights into the progression of health and disease over a lifetime. Globally, several national birth cohort studies such as the UK Millennium Cohort Study, the Norwegian Mother, Father, and Child Cohort Study (MoBa), and the Danish National Birth Cohort, have advanced our understanding of early‐life health determinants [2, 3, 4]. In China, birth cohort research began in 1993 with studies on folic acid and neural‐tube defects prevention [5]. The China Birth Cohort study later recruited pregnant women across 17 provinces to investigate factors related to birth defects, but ended follow‐up at birth [6]. Other cohorts, like the Born in Guangzhou Cohort Study [7], Shanghai Birth Cohort [8], Jiaxing Birth Cohort [9], Xi'an Longitudinal Mother‐Child Cohort [10], and China‐Anhui Birth Cohort Study [11], have been constructed to target cities with high to mid‐level socioeconomic development in eastern and southern China, leaving western regions underrepresented. Although the China Southwest Birth Cohort included parts of the Western China, the enrollment was limited to Sichuan, Chongqing, and Guizhou [12].

Western China, which comprises approximately 70% of the country's landmass with nearly one‐third of its population, presents unique health challenges due to geographical, environmental, and socioeconomic diversity [13]. Compared to the eastern regions, Western China faces greater disparities in healthcare access, lifestyle factors, genetic predispositions, and environmental exposures that may influence fetal development, birth outcomes, and early childhood health [14]. One of the distinctive features of Western China is its high‐altitude environment, where hypobaric hypoxia may contribute to fetal growth restriction, pregnancy complications, and neonatal hypoxia [15, 16]. Besides, the diverse ethnic populations in Western China, each with distinct genetic backgrounds, cultures, and dietary habits, further contribute to variations in maternal‐child health outcomes [14]. Despite these unique factors, Western China remains underrepresented in large‐scale birth cohort studies, limiting the ability to develop effective interventions and prevention strategies tailored to this population.

To address these gaps, we launched the Western China Birth Cohort (WCBC) study, a multicenter, longitudinal, and large‐scale prospective study. From October 2020 to February 2025, WCBC had enrolled a total of 15,093 pregnant women from five provinces in Western China, including Chongqing, Gansu, Guizhou, Qinghai, and Sichuan, across eight clinical centers, spanning a vast range of altitudes and socioeconomic settings. The recruitment will continue until 100,000 mother‐child dyads are enrolled. A total of 12 different types of biological samples have been collected in the study from mother or child, including peripheral blood, cord blood, dried blood spots, placenta, umbilical cord, decidua, saliva, fecal samples, throat and nasal swabs, vaginal swabs, and breast milk. As the first large‐scale birth cohort in Western China, the WCBC study will provide valuable insights into early‐life determinants of health and diseases across diverse populations, informing public health strategies and clinical approaches for Western China and also regions with similar geographical and socio‐cultural contexts.

## STUDY DESIGN

### Recruitment

**Figure 1:**
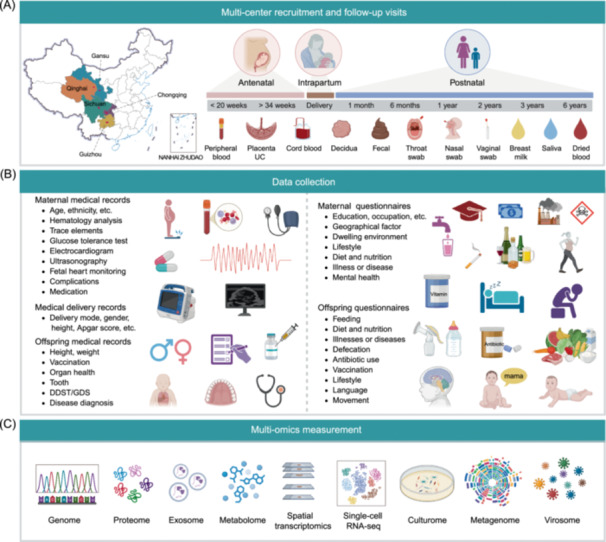
Study design of the WCBC cohort. (A) Multi‐center recruitment and follow‐up visits. (B) Collection of medical records and questionnaires. (C) Multi‐omics measurement. DDST, Denver Developmental Screening Test; GDS, Gesell Developmental Schedules; UC, umbilical cord; WCBC, Western China Birth Cohort.

Figure 1 summarizes the study design. WCBC study enrolled pregnant women from eight Tertiary A‐rated maternal and child healthcare hospitals across five provinces in Western China, including Chongqing, Gansu, Guizhou, Qinghai, and Sichuan (Figure 1A and Table S1). All participating clinical centers maintain standardized facilities for biological sample processing and storage. The geographic distribution of participants included a wide range of altitudes (42–4805 m) and terrains. Qinghai is located on the Qinghai‐Tibet Plateau with an average altitude of 3000 m. Gansu is at the edge of the Qinghai‐Tibet Plateau, with an average altitude of 2200 m. Sichuan has a diverse and complex terrain. Its western part consists of numerous mountains extending from the Qinghai‐Tibet Plateau, while its eastern region lies within the fertile Sichuan basin. Chongqing, adjacent to eastern Sichuan, is dominated by mountains with an average altitude of 400 m. Guizhou, located at the eastern end of the Yungui Plateau, has higher altitudes in its western and central regions.

Eligible participants were pregnant women who (1) were under 20 weeks of gestation and intended to attend routine antenatal examinations and give birth at our designated clinical centers; (2) had no plans to relocate within the next 6 years; (3) had adequate reading and communication ability to understand the study; (4) were willing to sign the informed consent; (5) maintained a stable phone number for the next 6 years. Pregnant women were excluded if they had cognitive impairment, psychiatric disorders, or insufficient reading abilities. Participants could withdraw from the study at any stage. The study was approved by the Ethics Committee of the Children's Hospital of Chongqing Medical University and other participating hospitals (No. 4‐5/2020 and 260‐1/2023) and was registered with the Chinese Clinical Trial Registry (ChiCTR2000038372, https://www.chictr.org.cn/showproj.html?proj=61336).

### Follow‐up visits and data collection

The WCBC included nine scheduled follow‐up visits throughout the study: early or middle pregnancy (<20 weeks), late pregnancy (>34 weeks), delivery, and postnatal follow‐ups at 1 month, 6 months, 1 year, 2 years, 3 years, and 6 years. At each visit, biological samples, clinical data, and questionnaires were systematically collected following standardized protocols (Figure 1A,B and Table 1). Pregnant women were recruited during early or mid‐pregnancy at designated clinical centers. Eligibility was assessed based on predefined inclusion criteria, and those who provided informed consent were enrolled. Upon enrollment, participants' demographic information, medical records (e.g., laboratory tests, imaging data, delivery records, and child healthcare records), and questionnaire data were continuously documented throughout the study (Table 1; see “Collection of Clinical Data” and “Collection of Questionnaires” in the Supporting Information). Structured questionnaires were administered at multiple follow‐up points to collect longitudinal data on maternal health, socioeconomic status, environmental exposures, lifestyle factors, and child development. To assess cognitive and motor development, developmental screening and evaluation tools, including the Denver Developmental Screening Test (DDST) and Gesell Developmental Schedules (GDS), were incorporated.

**Table 1:** Maternal and child data collection in WCBC.

Measurements	Antenatal			Postnatal					
	<20 weeks	>34 weeks	Delivery	1 month	6 months	1 year	2 years	3 years	6 years
**Mother**									
**Questionnaire**									
Baseline demographics	√								
Medical history	√								
Maternity history	√								
Menstrual cycle	√								
Family medical history	√								
Dwelling environment	√								
Lifestyle	√	√							
Illnesses or diseases	√	√							
Diet and nutrition	√	√			√				
Mental health	√	√		√	√	√	√	√	
**Medical records**									
Hematology analysis	√	√							
Urinalysis	√	√							
Imaging examination	√	√							
Delivery information			√						
**Biological samples**									
Peripheral blood	√	√							
Fecal	√	√		√	√	√			
Throat swab	√	√	√	√	√	√			
Nasal swab	√	√	√	√	√	√			
Saliva	√	√	√	√	√	√			
Vaginal swab			√						
Placenta			√						
Umbilical cord			√						
Decidua			√						
Breast milk				√	√	√			
**Child**									
**Questionnaire**									
Feeding				√	√	√			
Diet and nutrition				√	√	√	√	√	√
Illnesses or diseases				√	√	√	√	√	√
Defecation					√	√	√	√	√
Medications					√	√	√	√	√
Vaccination				√	√	√	√	√	√
Lifestyle					√	√	√	√	√
Neurodevelopment				√	√	√	√	√	√
Language				√	√	√	√	√	√
Movement				√	√	√	√	√	√
**Medical records**									
Basic characteristics			√						
Birth defect/diseases			√						
Physical measurements				√	√	√	√	√	√
DDST/GDS				√	√	√	√	√	√
**Biological samples**									
Cord blood			√						
Dried blood spot			√						
Capillary blood				√					
Peripheral blood					√	√	√		
Meconium			√						
Fecal				√	√	√			
Throat swab				√	√	√			
Nasal swab				√	√	√			
Saliva				√	√	√			

Abbreviations: DDST, Denver Developmental Screening Test; GDS, Gesell Developmental Schedules; WCBC, Western China Birth Cohort.

## BIOLOGICAL SAMPLING PROCEDURES

Biological samples were collected at designated follow‐up visits, as outlined in Table 1. Blood samples, including whole blood, plasma, white blood cells, red blood cells, cord blood, and dried blood spots, were the major biological samples collected in WCBC (see “Biological sampling procedures” in the Supporting Information). To minimize unnecessary procedures, blood samples from children were only collected when surplus blood was available after clinical testing. In addition to blood samples, placenta, umbilical cord, decidua, fecal samples, throat and nasal swabs, saliva, vaginal swabs, and breast milk were collected from participants at the Chongqing clinical center. To ensure sample integrity and minimize batch effects, all participating medical centers adhered to the same SOPs for sample collection, pre‐processing, transportation, and storage. Multi‐omics analyses were designed based on the biological samples collected, enabling studies in genomics, proteomics, exosome profiling, metabolomics, spatial transcriptomics, single‐cell RNA sequencing, microbiome analysis (culturome and metagenomics), and virosome profiling (Figure 1C, see “Multi‐omics measurement” and “Statistical analysis” in the Supporting Information).

## PROGRESS OF THE COHORT

**Figure 2:**
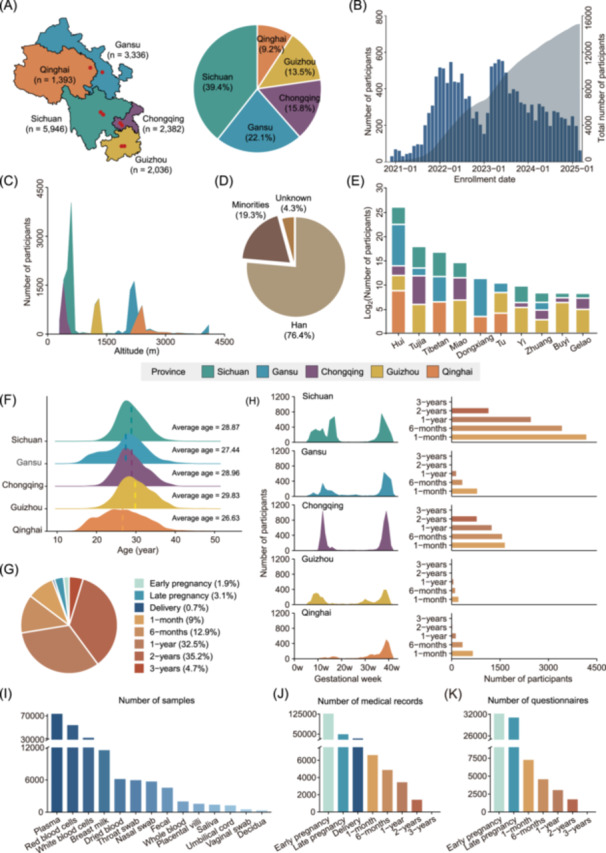
The baseline characteristics of participants. (A) Geographic distribution of participants across provinces. (B) Monthly enrollment of participants. (C) Distribution of altitudes for participants from different provinces. (D) A pie chart showing the proportions of Han and ethnic minority participants. (E) The top ten ethnic minority groups in WCBC. (F) Distribution of pregnant age of participants from different provinces. The dashed line represents the average age of pregnant women in each province. (G) Proportions of participants who reached each follow‐up visit. (H) Numbers of participants who completed each follow‐up visit. Number of biological samples (I), medical records (J), and questionnaires (K).

From October 2020 to February 2025, WCBC enrolled 15,093 pregnant women, including 5946 from Sichuan, 3336 from Gansu, 2382 from Chongqing, 2036 from Guizhou, and 1393 from Qinghai (Figure 2A and Table S1). On average, 77 participants were recruited per month in WCBC (Figure 2B and Table S2). Participants were distributed across high‐altitude (Qinghai and Gansu), middle‐altitude (Guizhou), and low‐altitude areas (Sichuan and Chongqing) (Figure 2C and Table S3). The distributions of baseline maternal BMI, education level, occupation, marital status, and the yearly family income are shown in Figure S1A–E. More than 76% (11,525) of the participants were of Han ethnicity, with 19.3% (2913) belonging to minority ethnic groups (Figure 2D). The top ten ethnic minorities included Hui, Tujia, Tibetan, Miao, Dongxiang, Tu, Yi, Zhuang, Buyi, and Gelao (Figure 2E and Table S4). Women from high‐altitude areas conceived earlier than those from middle and low‐altitude areas (Kruskal–Wallis test *p* value < 2.2E‐16, Figure 2F and Table S5). 93.9% (14,171) of the participating women had given birth, and their children had reached 2 to 3 years of age (Figure 2G and Table S6). Figure 2H and Table S7 illustrated the number of participants who completed each follow‐up visit, reflecting the ongoing retention across scheduled visits. On average, prenatal follow‐ups were conducted at 10–16 and 32–38 weeks of gestation, with Qinghai participants often recruited later due to delayed access to pregnancy care. Offspring follow‐ups have been conducted from 1 month to 3 years of age. The major pregnancy complications of participants are shown in Figure S1F. The three most frequent categories were endocrine, nutritional, and metabolic diseases; blood and immune system diseases; and amniotic fluid, fetal membrane, and placenta disorders. The gestational ages and birth weights of newborns are shown in Figure S1G,H.

By February 2025, WCBC had collected more than 200,000 biological samples, including 73,393 plasma samples, 54,302 red blood cell samples, 33,196 white blood cell samples, 11,536 breast milk samples, 6,166 dried blood spot samples, 5956 throat swabs, 5701 nasal swabs, 4534 fecal samples, 1988 whole blood samples, 1559 placental villus, 1389 saliva samples, 1254 umbilical cord samples, 518 vaginal swabs, and 352 decidua samples (Figure 2I). In addition, over 220,000 medical records and 80,000 questionnaires were collected, forming a valuable resource for maternal and child health research in Western China (Figure 2J,K and Tables S8, S9).

## DISCUSSION

The WCBC is a large‐scale, multi‐center, longitudinal birth cohort study designed to investigate maternal and child health in Western China. A key strength of WCBC is its geographical and ethnic diversity, offering a unique opportunity to investigate altitude‐related and ethnicity‐specific influences on maternal and child health. Spanning from low‐altitude regions to the high‐altitude Qinghai‐Tibet Plateau, the cohort allows for the study of how hypoxia, temperature extremes, and ultraviolet radiation affect pregnancy outcomes, fetal growth, and early childhood development, as these environmental factors may contribute to variations in birth weight, preterm birth risk, and neonatal complications [15, 17, 18]. In addition, WCBC is among the first large‐scale birth cohorts in China to include a substantial ethnic minority population, with 19.3% of participants representing such as Hui, Tujia, Tibetan, Miao, and Dongxiang communities. This diversity enables investigations into ethnicity‐specific health risks, genetic predispositions, dietary patterns, and cultural practices that may influence maternal and child health outcomes, contributing to a broader understanding of population‐specific determinants of health in multi‐ethnic settings.

Another notable aspect of WCBC is its comprehensive follow‐up strategy, which spans nine scheduled visits from early pregnancy (<20 weeks) through 6 years of childhood. This longitudinal design captures critical developmental periods spanning the perinatal and early childhood development, allowing for the systematic evaluation of fetal development, postnatal growth, neurodevelopment, and disease risk trajectories, providing insights into the early‐life risk factors for multiple diseases. Furthermore, WCBC integrates extensive biological sampling, enabling multi‐omics profiling across different stages of development. This includes genomics, proteomics, exosome profiling, metabolomics, spatial transcriptomics, single‐cell RNA sequencing, microbiome, and virosome profiling, establishing a valuable platform for studying gene‐environment interactions and molecular mechanisms underlying maternal and child health and disease conditions. Additionally, the timing of WCBC recruitment during the COVID‐19 pandemic presented a unique opportunity to investigate the impact of SARS‐CoV‐2 infection on pregnancy outcomes, fetal development, and early childhood immunity.

Some limitations of this cohort study must be acknowledged. Firstly, we experienced a relatively high rate of loss to follow‐up (18%) at the beginning due to the COVID‐19 pandemic. Secondly, many families transitioned from delivery hospitals to community hospitals for ongoing pediatric healthcare. To address this challenge, collaborations with community hospitals were established to retrieve child growth and developmental data and ensure successful follow‐up completion.

## CONCLUSION

In conclusion, the WCBC study establishes a valuable resource for advancing research on maternal and child health in Western China, integrating clinical data, questionnaires, and multi‐omics analyses with comprehensive biological sampling. This cohort will provide critical insights into altitude‐related pregnancy outcomes, ethnicity‐specific health risks, and early‐life developmental trajectories, contributing to a better understanding of disease susceptibility, growth patterns, and long‐term health outcomes in this diverse population. Findings from WCBC will support the development of clinical interventions and public health strategies to improve maternal and child health in Western China and other regions with similar environmental and socio‐cultural settings worldwide.

## AUTHOR CONTRIBUTIONS

**Xiangyu Li**: Formal analysis; visualization; writing—original draft; writing—review and editing; methodology; investigation. **Ying Wu**: Formal analysis; writing—original draft; visualization; validation. **Bin Yi**: Formal analysis; writing—original draft; visualization; validation. **Mengjie Chen**: Formal analysis; visualization; writing—original draft; writing—review and editing. **Gang Zhang**: Formal analysis; visualization; writing—review and editing; writing—original draft. **Xiaoshan Shao**: Formal analysis; visualization; writing—review and editing; writing—original draft. **Xiulian Jiang**: Formal analysis; visualization; writing—review and editing; writing—original draft. **Yuxia Cui**: Formal analysis; visualization; writing—review and editing; writing—original draft. **Li Chen**: Data curation; resources. **Xiaojing Dong**: Data curation; resources. **Shu Zhang**: Data curation; resources. **Yao Zhao**: Data curation; resources. **Yuebi Deng**: Data curation; resources. **Xueqi Li**: Visualization; software; formal analysis. **Yang Wang**: Software; formal analysis; visualization. **Lei Wu**: Software; visualization. **Yu Fu**: Software; visualization. **Dan Ran**: Software; visualization. **Chen Peng**: Software; data curation. **Xiao Yang**: Software; data curation. **Lan Zhang**: Data curation; resources. **Yanxia Wang**: Data curation; resources. **Yi Zhu**: Data curation; resources. **Dina Sun**: Data curation; resources. **Yuchen Ran**: Data curation; resources. **Dan Zheng**: Data curation; resources. **Xuan Yin**: Data curation; resources. **Yufen Chen**: Data curation; resources. **Yu Long**: Data curation; resources. **Wenjing Wang**: Data curation; resources. **Xiaodong Zhao**: Conceptualization; supervision. **Enmei Liu**: Conceptualization; funding acquisition; resources; writing—review and editing. **Tao Xu**: Conceptualization; funding acquisition; writing—review and editing; resources. **Qiu Li**: Conceptualization; funding acquisition; writing—review and editing; resources; project administration. **Wen Zhong**: Conceptualization; funding acquisition; visualization; resources; project administration; investigation; writing—original draft, review and editing.

## CONFLICT OF INTEREST STATEMENT

The authors declare no conflicts of interest.

## Supporting information


**Figure S1.** Demographic and clinical characteristics of participants in WCBC.

**Table S1.** Clinical centers participating in the WCBC study.
**Table S2.** Monthly enrollment of participants in the WCBC study.
**Table S3.** Geographic and altitudinal distribution of enrolled participants.
**Table S4.** Numbers of participants from the top 10 minority ethnic groups with the highest enrollment.
**Table S5.** Age distribution of pregnant women enrolled in the WCBC study.
**Table S6.** Distribution of participants across follow‐up visits.
**Table S7.** Numbers of participants completing scheduled follow‐up at each visit.
**Table S8.** Numbers of medical records collected at each follow‐up visit.
**Table S9.** Numbers of questionnaires collected at each follow‐up visit.

## Data Availability

The data that support the findings of this study are available from the corresponding author upon reasonable request. The datasets generated during the WCBC study have been securely deposited at the Children's Hospital of Chongqing Medical University (http://wcbc.chcmu.com/), in full compliance with ethical regulations and patient confidentiality agreements. Due to ethical and legal restrictions, participant‐level data are available for research and scientific purposes only upon request, subject to approval by the Ethics Committee of the Children's Hospital of Chongqing Medical University and execution of appropriate data transfer agreements. This includes submitting a research proposal to the corresponding author or sent to chcmu_wcbc@163.com, where upon approval, all data analysis need to be done on a local server with protected access, in compliance with Personal Information Protection Law of the People's Republic of China (PIPL) and Regulations on the Administration of Human Genetic Resources of the People's Republic of China. The aggregated data and scripts used in the study are saved in Github (https://github.com/WenZhong-Lab/BirthCohort). Supplementary materials (figures, methods, tables, graphical abstract, slides, videos, Chinese translated version, and update materials) may be found in the online DOI or iMeta Science http://www.imeta.science/.
