# Supplementary material for: A multi‐centered prospective birth cohort study in Western China
Source: Imeta. 2025 May 26;4(3):e70049. doi: 10.1002/imt2.70049 (PMC12130564; doi:10.1002/imt2.70049)
Supplement: Supplementary file 1 — Figure S1. Demographic and clinical characteristics of participants in WCBC. [file IMT2-4-e70049-s002.docx]

**Supporting information to**

**A multi-centered prospective birth cohort study in Western China**

**Running title:** Longitudinal birth cohort in Western China

Xiangyu Li^1,2#^, Ying Wu^3#^, Bin Yi^4#^, Mengjie Chen^5#^, Gang Zhang^6,7#^, Xiaoshan Shao^8#^, Xiulian Jiang^9#^, Yuxia Cui^10#^, Li Chen^11^, Xiaojing Dong^5^, Shu Zhang^1^, Yao Zhao^1^, Yuebi Deng^1^, Xueqi Li^2^, Yang Wang^1^, Lei Wu^1^, Yu Fu^1^, Dan Ran^1^, Chen Peng^2^, Xiao Yang^3^, Lan Zhang^3^, Yanxia Wang^4^, Yi Zhu^5^, Dina Sun^5^, Yuchen Ran^12^, Dan Zheng^13^, Xuan Yin^13^, Yufen Chen^9^, Yu Long^10^, Wenjing Wang^10^, Xiaodong Zhao^14^, Enmei Liu^15*^, Tao Xu^2*^, Qiu Li^16*^, Wen Zhong^1,2*^

^1^ Department of Early Life Development and Cohort Research Laboratory, Children's Hospital of Chongqing Medical University, National Clinical Research Center for Child Health and Disorders, Ministry of Education Key Laboratory of Child Development and Disorders, ﻿Chongqing Municipal Health Commission Key Laboratory of Children's Vital Organ Development and Diseases, Chongqing 401122, China

^2^ Guangzhou National Laboratory, Guangzhou 510005, China

^3^ Chengdu Women's and Children's Central Hospital, School of Medicine, University of Electronic Science and Technology of China, Chengdu 611731, China

^4^ Gansu Provincial Maternity and Child-Care Hospital, Gansu Provincial Pediatric Clinical Research Center, Lanzhou 730050, China

^5^ The Second Affiliated Hospital of Chongqing Medical University, Chongqing 400000, China

^6^ Department of Obstetrics, Sichuan Provincial Women's and Children's Hospital, The Affiliated Women's and Children's Hospital of Chengdu Medical College, Chengdu 610045, China

^7^ Department of Hepatobiliary and Vascular Surgery, The First Affiliated Hospital of Chengdu Medical College, Chengdu 610500, China

^8^ Department of Renal Rheumatology and Immunology, Guiyang Maternal and Child Health Care Hospital, Guiyang 550003, China

^9^ Qinghai Provincial Women and Children's Hospital, Xining 810007, China

^10^ Shanghai Children's Medical Center Guizhou Hospital, Shanghai Jiao Tong University School of Medicine, GuiYang 550081, China

^11^ Growth, Development and Mental Health Center of Children and Adolescents, Chongqing Key Laboratory of Child Neurodevelopment and Cognitive Disorders, National Clinical Research Center for Child Health and Disorders, Ministry of Education Key Laboratory of Child Development and Disorders, Children’s Hospital of Chongqing Medical University, Chongqing 400014, China

^12^ Department of Child Healthcare, Sichuan Provincial Women's and Children's Hospital, The Affiliated Women's and Children's Hospital of Chengdu Medical College, Chengdu 610045, China

^13^ Department of Maternity Health, Guiyang Maternal and Child Health Care Hospital, Guiyang 550003, China

^14^ Department of Rheumatism and Immunology, Children's Hospital of Chongqing Medical University, National Clinical Research Center for Child Health and Disorders, Ministry of Education Key Laboratory of Child Development and Disorders, Chongqing Key Laboratory of Child Rare Diseases in Infection and Immunity, Chongqing 401122, China

^15^ Department of Respiratory Medicine, Children's Hospital of Chongqing Medical University, National Clinical Research Center for Child Health and Disorders, Ministry of Education Key Laboratory of Child Development and Disorders, Chongqing Key Laboratory of Child Rare Diseases in Infection and Immunity, Chongqing 401122, China

^16^ Department of Nephrology, Children’s Hospital of Chongqing Medical University, National Clinical Research Center for Child Health and Disorders, Ministry of Education Key Laboratory of Child Development and Disorders, Chongqing Key Laboratory of Pediatric Metabolism and Inflammatory Diseases, Chongqing 401122, China

^#^These authors contributed equally: Xiangyu Li, Ying Wu, Bin Yi, Mengjie Chen, Gang Zhang, Xiaoshan Shao, Xiulian Jiang, and Yuxia Cui

*Correspondence: [zhong_wen@gzlab.ac.cn](mailto:zhong_wen@gzlab.ac.cn) (Wen Zhong), [liqiu809@126.com](mailto:liqiu809@126.com) (Qiu Li), [xutao@ibp.ac.cn](mailto:xutao@ibp.ac.cn) (Tao Xu), [emliu186@126.com](mailto:emliu186@126.com) (Enmei Liu)

**Supplementary methods**

**Collection of clinical data**

Upon enrollment during early or middle pregnancy (< 20 weeks), each pregnant women completed a baseline clinical assessment and questionnaire. Follow-up visits were scheduled for late pregnancy (> 34 weeks) and at delivery. Depending on the electronic medical record system, we collected the maternal basic information (e.g. age, ethnicity and height), hematological tests (e.g. complete blood count, liver markers, kidney markers, and glucose tolerance test), urinalysis, electrocardiogram, ultrasonography, fetal heart monitoring, complications, and medication at early or middle and late pregnancy visits (Table 1).

Hematological test included the following biological indicators: 1) Complete blood count: white blood cells (WBC), red blood cells (RBC), hemoglobin (HGB), hematocrit (HCT), platelet count (PLT), mean platelet volume (MPV), mean corpuscular volume (MCV), mean corpuscular hemoglobin (MCH), neutrocyte (NEUT), lymphocyte (LYMPH), monocyte (MONO), eosinocyte (EO), and basophilic granulocyte (BASO); 2) Liver biomarkers: alanine transaminase (ALT), aspartate Transaminase (AST), alkaline phosphatase (ALP), lactate dehydrogenase (LDH), gamma-glutamyltransferase (GGT), total bile acids (TBA), and albumin (ALB); 3) Kidney biomarkers: blood urea nitrogen (BUN), creatinine (CREA), uric acid (UA), retinol binding protein (RBP), and cystatin C (CysC); 4) Blood lipids: total cholesterol (TC), triglyceride (TG), low-density lipoprotein (LDL), and how-density lipoprotein (HDL); 5) Trace elements: Ca, Cu, Zn, Mg, Fe, and Pb; 6) Oral glucose tolerance test: fasting blood glucose (OGTT-0h), blood glucose at 1hour (OGTT-1h), and blood glucose at 2 hours (OGTT-2h). Urinalysis included the following biological indicators: acidity (pH), specific gravity (SG), blood (BLD), glucose (GLU), protein (PRO), ketones (KET), WBC, RBC, epithelial cells (EC), nitrites (NTT), bilirubin (BIL) and urobilinogen (URO).

Information on newborns’ basic characteristics at birth, including date of birth, delivery mode, gestational age, gender, Apgar score [1], weight, height, head circumference, and any birth defects/diseases, was obtained from the delivery records (Table 1). Routine health checks for children were conducted at 1 month, 6 months, 1 year, 2 years, 3 years and 6 years of age, at either the delivery hospital or community hospital. These checks mainly included physical measurements of height, weight, head circumference, bregmatic fontanel, as well as dental examinations, breast/testis volume, and evaluations of neurobehavioral, movement, and language development. The Denver Developmental Screening Test (DDST) [2] or the Gesell Development Schedules (GDS) [3] was conducted if a child was suspected to have a developmental disorder. Disease diagnoses were obtained from clinical records.

**Collection of questionnaires**

Participants completed questionnaires using the WCBC mobile app. Table 1 showed the questionnaires across different visits. At recruitment, a baseline questionnaire had been designed to collect maternal basic information (e.g. maternal education, occupation, family income, marital status, terrain of location, medical history, maternity history, menstrual cycle, and family medical history) and dwelling environment (e.g. water sources, toxic chemical exposure, decoration, and pets). Follow-up questionnaires during early or middle pregnancy (< 20 weeks) and late pregnancy (> 34 weeks) collected information on lifestyle factors (e.g. smoking, alcohol, physical activity, sleeping and use of electronic devices), illness and diseases, diet and nutrition supplements (e.g. vitamins, folic acid, omega-3, iron, calcium, and zinc) and mental health. Mental health assessments, using Patient Health Questionnaire-9 (PHQ-9) [4] for depression and Generalized Anxiety Disorder-7 (GAD-7) [5] for anxiety, were conducted at both early or middle pregnancy, late pregnancy and each of the postanal follow-up visits.

In addition, Information about children’s feeding (e.g. breastfeeding or formula feeding) was collected via questionnaires at 1, 6 and 12 months of age (Table 1). Children’s diet and nutrition supplements, illnesses and diseases, defecation, medication, vaccination, lifestyle information (e.g. eating behavior, sleeping, outdoor activities, and use of electronic devices), neurobehavioral development, and language and movement were collected via questionnaires at each of the child follow-up visits.

**Biological sampling procedures**

Maternal peripheral blood samples were collected at the early or middle pregnancy and late pregnancy. Cord blood samples were collected at delivery. To minimize unnecessary procedures, blood samples from children were only collected when surplus blood was available after clinical testing. The blood samples were transported under 4℃. For maternal peripheral blood and cord blood samples, 1ml of whole blood samples from late pregnancy and cord blood were directly stored at -80℃ before centrifugation, respectively. The remained whole blood samples were centrifuged for 10 minutes at 3000 rpm and 4℃, and separated into three layers: plasma, white blood cells (buffy coat) and red blood cells, which were aliquoted and extracted and stored at -80℃. The dried blood spots are stored at -20℃.

Placenta and umbilical cord samples were fresh-frozen in optimal cutting temperature solution (OCT), transported in dry ice and stored at -80℃. Throat swab, nasal swab, saliva, vaginal swab samples were transported under 4℃ and stored at -80℃ until further processing. The research nurse allocated a fecal collection package to participants at each visit in clinical center. Participants collected fecal specimens at home and placed in the Anaerobic Microbiome Collection Kit and DNA/RNA Shield Fecal Collection Tube in this package, respectively. The packages were delivered to biobank under 4℃ and stored at -80℃ until further processing. Breast milk samples were collected when a mother accompanied her child for routine healthcare check at designated clinical centers, which were transported under 4℃. Breast milk samples were centrifuged for 10 minutes at 680g and 4℃ and then the fatty layer was removed. The whey was centrifuged again for 30 minutes at 10000g and 4℃ and the fatty layer was removed again. Whey samples were aliquoted and extracted and stored at -80℃.

**Multi-omics measurement and data analysis**

Sample selection for multi-omics analyses will be based on sample type availability, preservation quality, participant phenotype diversity, and representation across different geographic and clinical subgroups to ensure robust and generalizable molecular profiling. Specifically, a stratified random sampling with post-stratification weighting will be employed to ensure adequate statistical power across altitude gradients and ethnic subpopulations. Power analysis will be performed for each omics modality to determine appropriate sample size and time points for detecting biologically meaningful differences between groups after controlling for multiple testing (FDR correction). Propensity score matching will be implemented to balance confounding variables, including maternal age, altitude, geographic location, ethnic group, and baseline characteristics (e.g. BMI), minimizing selection bias in downstream analyses.

The whole blood samples will be used for the whole-genome sequencing by the DNBSEQ-T7RS platform. Plasma and whey samples will be used for proteomics, extracellular vesicle (EV) surface proteomics and metabolomics measurement. Protein expression profiling will be conducted using the Olink Explore 3072 platform based on the technology named Proximity Extension Assay (PEA) technology. This platform enables the quantification of more than 3000 proteins with only 10 ul plasma with a high sensitivity, especially for the low expressed proteins [6,7]. EV counts and single-EV surface protein expression will be evaluated by Proximity Barcoding Assay (PBA) technology [8]. Untargeted metabolite profiling will be performed using ultraperformance liquid chromatography-tandem mass spectroscopy [9]. Placenta samples will be analyzed using spatial transcriptomics by the STOmics Stereo-seq platform [10]. Decidua tissues will be analyzed using single-nucleus RNA-seq by 10x Genomics platform. Fecal and vaginal swab samples will be used for metagenomics sequencing and bacterial culturomics [11,12]. Throat swab, nasal swab, and saliva samples will be used for virusomics. For metagenomics and virusomics, sequencing will be performed by using the Illumina NovaSeq 6000 platform.

All data analysis was conducted using the R programming environment (version 4.4.3), utilizing packages including dplyr, tibble, tidyr, reshape2, and Matrix for data maniputation, and ggplot2, ggsci, ggridges, ggspatial, ggforce, patchwork, ggbump, ggbreak, and geoviz for data visualization. The Kruskal–Wallis test was applied to assess differences of maternal ages between groups.

**REFERENCES**

1. Apgar, V. 1953. “A proposal for a new method of evaluation of the newborn infant.” *Current Researches in Anesthesia and Analgesia* 32: 260−267. <https://www.ncbi.nlm.nih.gov/pubmed/13083014>

2. Frankenburg, W K, J B Dodds. 1967. “The Denver developmental screening test.” *Journal of Pediatrics* 71: 181−191. <https://doi.org/10.1016/s0022-3476(67)80070-2>

3. Ball, R S. 1977. “The Gesell Developmental Schedules: Arnold Gesell (1880-1961).” *Journal of Abnormal Child Psychology* 5: 233−239. <https://doi.org/10.1007/BF00913694>

4. Kroenke, K, R L Spitzer, J B Williams. 2001. “The PHQ-9: validity of a brief depression severity measure.” *Journal of General Internal Medicine* 16: 606−613. <https://doi.org/10.1046/j.1525-1497.2001.016009606.x>

5. Spitzer, Robert L, Kurt Kroenke, Janet B W Williams, Bernd Lowe. 2006. “A brief measure for assessing generalized anxiety disorder: the GAD-7.” *Archives of Internal Medicine* 166: 1092−1097. <https://doi.org/10.1001/archinte.166.10.1092>

6. Zhong, Wen, Fredrik Edfors, Anders Gummesson, Goran Bergstrom, Linn Fagerberg, Mathias Uhlen. 2021. “Next generation plasma proteome profiling to monitor health and disease.” *Nature Communications* 12: 2493. <https://doi.org/10.1038/s41467-021-22767-z>

7. Zhong, Wen, Ozlem Altay, Muhammad Arif, Fredrik Edfors, Levent Doganay, Adil Mardinoglu, Mathias Uhlen, Linn Fagerberg. 2021. “Next generation plasma proteome profiling of COVID-19 patients with mild to moderate symptoms.” *EBioMedicine* 74: 103723. <https://doi.org/10.1016/j.ebiom.2021.103723>

8. Wu, Di, Junhong Yan, Xia Shen, Yu Sun, Mans Thulin, Yanling Cai, Lotta Wik, et al. 2019. “Profiling surface proteins on individual exosomes using a proximity barcoding assay.” *Nature Communications* 10: 3854. <https://doi.org/10.1038/s41467-019-11486-1>

9. Li, Xiangyu, Hong Yang, Han Jin, Hasan Turkez, Gurkan Ozturk, Hamdi Levent Doganay, Cheng Zhang, et al. 2023. “The acute effect of different NAD(+) precursors included in the combined metabolic activators.” *Free Radical Biology and Medicine* 205: 77−89. <https://doi.org/10.1016/j.freeradbiomed.2023.05.032>

10. Zang, Xupeng, Dan Zhang, Wenjing Wang, Yue Ding, Yongzhong Wang, Shengchen Gu, Yijun Shang, et al. 2024. “Cross-Species Insights into Trophoblast Invasion During Placentation Governed by Immune-Featured Trophoblast Cells.” *Advanced Science* 11: e2407221. <https://doi.org/10.1002/advs.202407221>

11. Poyet, M, M Groussin, S M Gibbons, J Avila-Pacheco, X Jiang, S M Kearney, A R Perrotta, et al. 2019. “A library of human gut bacterial isolates paired with longitudinal multiomics data enables mechanistic microbiome research.” *Nature Medicine* 25: 1442−1452. <https://doi.org/10.1038/s41591-019-0559-3>

12. Lagier, Jean-Christophe, Saber Khelaifia, Maryam Tidjani Alou, Sokhna Ndongo, Niokhor Dione, Perrine Hugon, Aurelia Caputo, et al. 2016. “Culture of previously uncultured members of the human gut microbiota by culturomics.” *Nature Microbiology* 1: 16203. <https://doi.org/10.1038/nmicrobiol.2016.203>


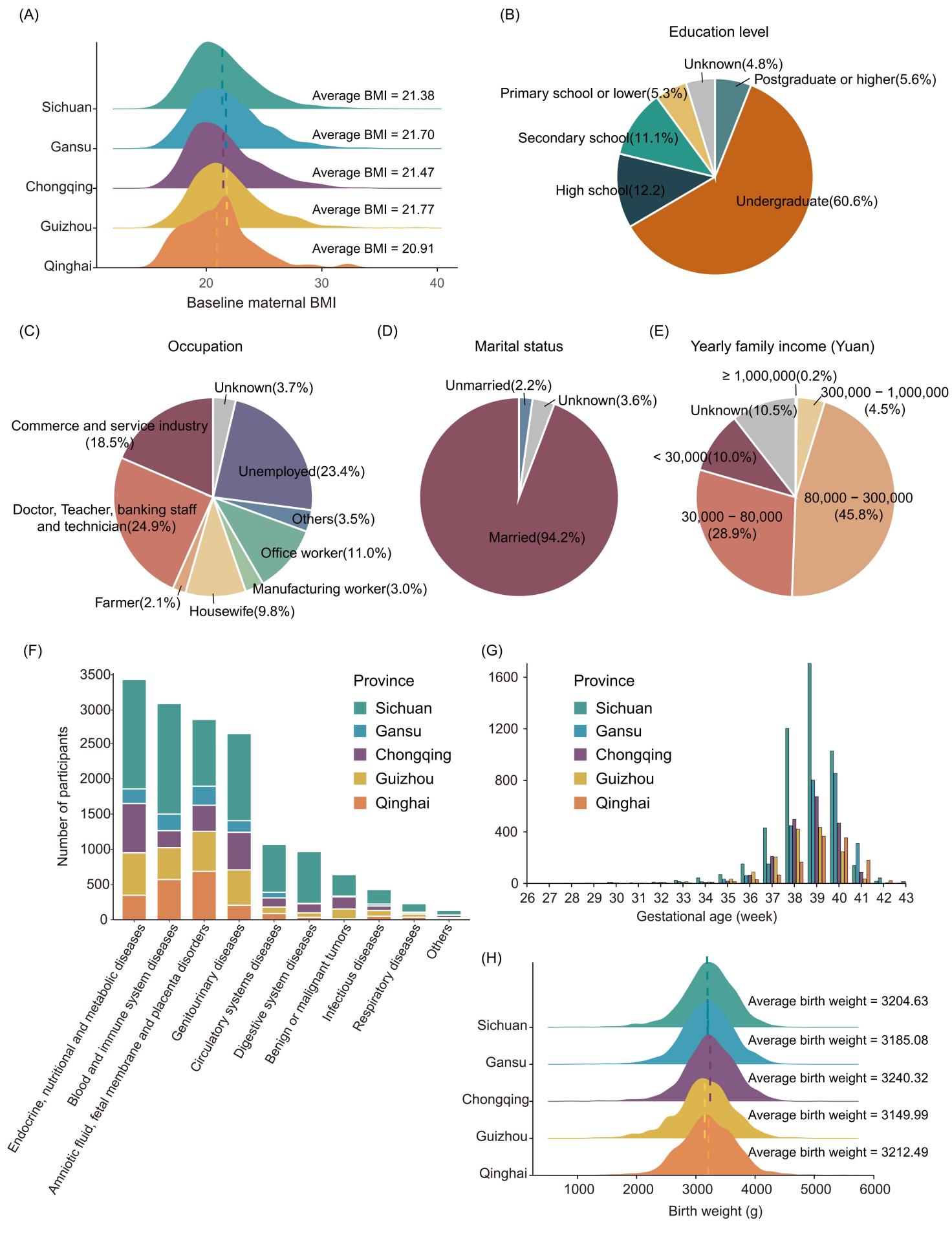


**Figure S1 Demographic and clinical characteristics of participants in WCBC.** (A-D) Distributions of baseline maternal BMI (A), education level (B), occupation (C), and marital status (D). (E) Distribution of yearly family income. (F) Distribution of major pregnancy complications. (G and H) Distribution of gestational ages (G) and birth weights (H) of newborns, color-coded by provinces. The dashed line represents the average BMI and birth weight in (A) and (H), respectively. WCBC: Western China Birth Cohort.
